# Kinematic Structure at the Early Flight Position in Ski Jumping

**DOI:** 10.2478/v10078-012-0077-6

**Published:** 2012-12-30

**Authors:** Janez Vodičar, Milan Čoh, Bojan Jošt

**Affiliations:** 1 University of Ljubljana, Faculty of Sport, Slovenia.

**Keywords:** ski jumping, kinematic analysis, early flight phase

## Abstract

The purpose of our research was to establish the variability of correlation between the length of the jumps and selected multi-item kinematic variables (n=9) in the early flight phase technique of ski jumping. This study was conducted on a sample of elite Slovenian ski jumpers (N=29) who participated in the experiment on a jumping hill in Hinterzarten, Germany (HS95m) on the 20^th^ of August, 2008. The highest and most significant correlations (p=0.01) with the length of the ski jump were found in the multi-item variable height of flying, which was also expressed with the highest level of stability of the explained total variance (TV) on the first factor (TV=69.13%). The most important characteristic of the aerodynamic aspect of early flight was the variable angle between the body chord and the horizontal axis with significantly high correlations (p<0.05). The stability of that aerodynamic factor was very high (TV=65.04%). The results were essentially similar for the multi-item variable angle between left leg and the horizontal axis (TV=61.88%). The rest of the multi-item kinematic variables did not have significant correlations with the multi-item variable length of jump. Only two more variables, the angle between the upper body and the horizontal plane (TV=53.69%), and the angle between left ski and left leg (TV=50.13%), had an explained common variance on the first factor greater than 50% of total variance. The results indicated that some kinematic parameters of ski jumping early flight technique were more important for success considering the length of the jump.

## Introduction

Many theoretical considerations and experimental approaches to the take-off and early flight of ski jumpers have been made, mostly from the biomechanical standpoint. The early flight phase is essential for the successful formation of the ski-jumping technique. The study of Arndt et al. (1995) reported very high multiple correlations (R2=.77) between five kinematic variables and length of the jump. In the early flight phase, the jumper has to reach the optimal height of the flying curve and maximise the horizontal velocity of the flight. During the take-off, a ski jumper has to solve five relatively independent motor tasks: the energy of the take-off, aerodynamics, accuracy, optimal angular momentum and arm movement (Vaverka, 1987). In many studies, the correlation coefficients between the biomechanical parameters describing the take-off and the length of jump occurred mainly in the interval r=0.40–0.60 (Vaverka et al., 1997). Some studies reported the importance of the optimal aerodynamic positions of the ski jumpers in the early flight phase (Janura et al., 2011; Jošt, 2010; Jošt et al., 1998; Schmölzer and Müller, 2005; Virmavirta et al., 2001; 2009; Vodičar and Jošt, 2011). The correlation between body angle and horizontal axis proved to be most significant (Virmavirta et al., 2005). The kinematic variables are the best predictor of the aerodynamic quality of flying. The relations could be explained from a biomechanical standpoint (Müller et al., 1996; Vaverka, 1987; Schwameder, 2008; Soong et al., 2004). The quality of the early flight position is dependent on more kinematic variables, which have to form an optimal combination for each jumper (Arndt et al., 1995).

The quality of early flight technique depends on many factors such as: the sports level of the ski jumpers, the characteristics of the jumping hill, in-run velocity, wind and weather conditions, etc. The best ski jumpers can perform early flight technique optimally in the most difficult conditions. In practice, we can find major differences in the quality of ski jumpers (Janura et al., 2011). The quality of early flight technique is determined in all repetitions by the same mechanical factors. The question is how those factors correlate to the length of the jump when the initial in-run conditions for the repetitions of jumps are similar, and when the repetitions are realised in a very short time with the same jumpers.

The first goal of this study was to establish a correlation between multi-item variable length of jumps and chosen multi-item kinematic variables in the early flight phase of ski jumping. The second goal was to determine the homogeneity of factor saturations on the first factor component. The third goal of this study consisted in the definition of the stability of the first factor component on each kinematic multi-item variable. According to previous studies of the early flight phase, it was expected that some multi-item kinematic variables could have significantly high correlations with the length of the jumps. In the case of significant levels of correlations with the multi-item variable length of jump (p=0.05) and their high level of homogeneity, a high level of explained common variance (TV) on the first explained factor (TV>50%) could be expected.

## Methods

The project was conducted on a sample of elite Slovenian ski jumpers (N=29) who participated in the experiment on the jumping hill in Hinterzarten, Germany (HS95m) on the 20^th^ of August, 2008. The jumpers provided informed written consent before the beginning of the experiment, which was approved by a local ethics committee and was performed according to the Helsinki Declaration. The jumpers performed seven jumps in two hours from the same in-run position. Wind was almost entirely absent, guaranteeing fairly equal conditions for the jumps. The dependent multi-item variable included the length of the jump (n=7), whereas the independent multi-item variables (n=7) were chosen from among other kinematic characteristics of ski jumping early flight technique. A SONY DSR 300-PK camera filmed the flight position at 15 m after the take-off table, operating at 50 fps, perpendicular to the sagittal plane (Picture 1).

The conditions on the jumping hill did not allow three-dimensional measurements, thus 2-D analysis in the sagittal plane was used. The image space was calibrated using calibration rods and markers along the observation area. The weather conditions were stable and all frames had good visibility. The image had a resolution of 720×567 pixels, i.e. a shift of the cursor by 1 pixel resulting in a linear resolution of 0.013 m. It was assumed that the maximal error of angle determination in this study was for a segment length of 0.55 m, at about 3.6 degrees. The precision limits for these angle measurements resulted predominantly from the inexactness in determining the ankle, hip and shoulder reference points; an athlete in his suit is not a rigid body. Associated with this are angle measurement precision errors of typically 1–2° (Schmölzer and Müller, 2005). A six-link bilateral model was created (left ski, right ski, trunk, arm, thigh, shin) based on nine joint points (top of the skis, end of the skis, shoulder joint, distal arm joint, hip joint, knee joint and ankle joint) (Picture 2).

**Figure 1:**
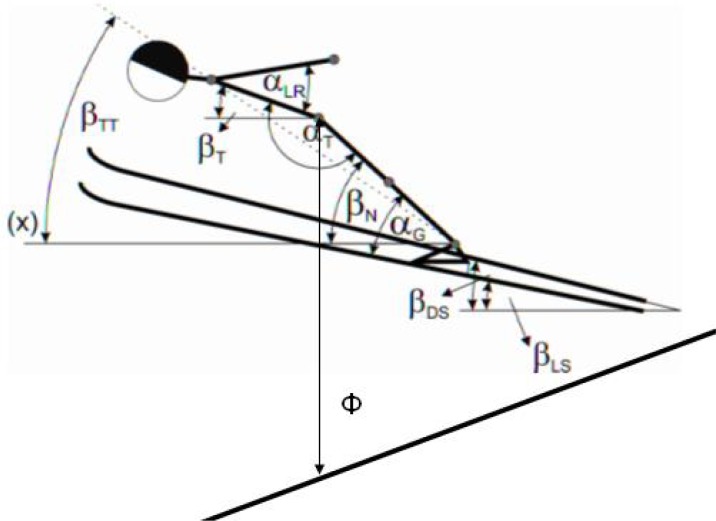
Set of kinematic variables at 15m behind the jumping hill edge; α _G_- Angle between left skis and leg; α_T_- Angle of hip extension; α_LR_- Angle between upper body and left arm; β_N_- Angle between left leg and horizontal axis; β_T_- Angle between upper body and horizontal axis; β_TT_- Angle between body chord and horizontal axis; β_LS_- Angle between left skis and horizontal axis; β_DS_- Angle between right skis and horizontal axis; Φ Vertical height of flying; x- horizontal axis

The data were manually digitised by an experienced technician. The changes of body and ski positions were mostly determined with respect to the horizontal plane. The set of eight kinematic variables was constructed (Figure 1).

Statistical analysis of all multi-item variables was performed to determine mean values (M) and standard deviations (SD). Pearson’s linear correlation coefficients (r) were computed. P-values of less than 0.05 were accepted as statistically significant. Factor component analysis was used to determine the common variance between the dependent multi-item variable length of jump and the chosen independent multi-item kinematic variables. The following parameters were calculated: F_n_p – factors value of each manifest variable on extracted factors, F _CUM_ – cumulative factors value of each manifest variable of all extracted factors, % of TV – percentage of total variance of all extracted factors.

## Results

All correlation coefficients between the dependent multi-item variable length of the jump and the independent multi-item variable vertical height of flying (Table 1) were statistically significant (p<0.05). High factor projections of both multi-item variables vertical height of flying and length of jump existed in the first common factor, which explained 69.13 % of total variance. Statistically significantl (p<0.05) coefficients of correlations between the multi-item variable angle between the body chord and horizontal axis and length of jump were reached. A high level of total variance (TV=65.04%) was seen in the first common factor. Also statistically significant correlation coefficients existed between the multi-item variable length of jump and the angle between the left leg and the horizontal axis. The variability of these coefficients was not high. The explained common variance (TV=61.88%) in the first factor was above 50 % of the total variance.

The correlation coefficients between the multi-item variable length of jump and angle between upper body and horizontal plane were not all significant. They ranged from r=0.12; p>0.05 to r=0.54; p<0.01. The common explained variance (TV=53.69%) of the first factor was greater than 50% of total variance (Table 2). More than 50% of total variance existed between the multi-item variable length of jump and angle between left skis and left legs. Correlation coefficients were no longer significantly high, and the variability of these coefficients was higher. Only one statistically significant correlation coefficient (r =0.39, p<0.05) was found in the sixth round.

The rest of the multi-item kinematic variables angle between the left skis and the horizontal axis, the angle between right skis and the horizontal axis, and the angle between upper body and the left arm had explained common variances of the first factor of less than 50% of total variance (Table 3). The coefficients of linear correlations showed statistically significant values.

## Discussion

The highest and most significant correlations with the length of the jump were found in the multi-item variable height of flying. The difference between the lowest correlation coefficient (r=0.43; p<0.05) and the highest (r=0.72; p<0.01) was unexpectedly large. The best ski jumpers had a greater height of flying curve at the point of 15 m after the take-off. This characteristic is a consequence of the take-off technique as the most important movement phase in ski jumping, because it determines the initial velocity of flying, the angle of early flying curve, the angular momentum of rotation of the body and the aerodynamic position of the ski jumper system during the flight (Virmavirta and Komi, 1994). Factor projections of the manifest variables were high and very homogeneous. The maximal value of the total variance (TV=69.13%) of the first common factor was determined. The height of flying in the early flight phase is strongly influenced by the vertical velocity at take-off. Vertical velocity at the end of the take-off table depends biomechanically on the take-off power in the contact phase of the take-off movement (Virmavirta and Komi, 1989; 1993; 1994). It is important that the take-off power is developed in the optimal time and at the optimal location on the take-off table. There could be significantly high coefficients of correlation between the precision of the take-off and the length of the jump (Vaverka, 1987).

The most important indicator of the aerodynamic flight was the multi-item variable angle between the body chord and the horizontal axis (X) of the body movement. The correlation coefficients for these multi-item variables were high and statistically significant in all jumping attempts. Better jumpers more quickly established the optimal aerodynamic position for the flight. Similar results were found in some studies by Virmavirta et al. (2005) and Schwameder et al. (2004) at the Olympic competition in 2002 on the jumping hills K120 and K90. This competence was a relatively stable technique feature of the motor movement of the jumpers. The amount of total variance (TV=65.04%) in the first common factor significantly exceeds the value of 50%. Factor weights of the manifest variables in this factor were high and nearly homogeneous. A smaller body chord angle relative to the horizontal axis during the early flight supports a more effective aerodynamic position in the early phase of flying (Janura et al., 2011; Müller et al., 1996).

Based on the factor weights in the first factor and the high level of explained variance (TV=61.88%), we can conclude that the best jumpers have a smaller angle between the left leg and horizontal axis. This allows for better aerodynamic efficiency (minimising the drag force in the leg). The minimum value of the angle is subject to the favourable position of the lower leg during the take-off in the contact take-off phase (Janura, 2011). Jumpers who placed their lower leg more in the anterior position could more efficiently carry out a rotation of the body at the take-off (common centre of gravity moved forward more quickly). This allowed them to use the take-off force more efficiently. Part of this force is given to the rotation of the body (Schmölzer and Müller, 2005). Favourable or optimal resolution of the rotation torque of the body is one of the most demanding motor tasks of the jumper. Errors in this motor characteristic of the jumpers can cause many difficulties in the ski-jumping technique. The favourable position of the lower leg in the contact take-off phase allows faster movement of the femur around the knee joint (greater angular velocity) and faster movement of the centre of gravity forward (Vaverka, 1987). This is one of the key characteristics of success in the early flight phase (Arndt et al., 1995; Janura et al., 2011).

Similar tendencies were also found at the multi-item variable angle between upper body and horizontal axis. Better jumpers are generally less open in their upper body and therefore minimise the value of drag force on the trunk. This characteristic is important in the initial phase of flight, because this feature allows the jumper to maintain the highest possible horizontal speed. In particular, this movement technique shows positive trends on the largest hills, where the speed is much higher. The optimal position of the upper body allows the jumpers in the first part of flight the highest possible aerodynamic efficiency and a faster transition to the optimal position for the middle phase of flying (Vaverka, 1987).

One important variable in aerodynamic terms in the first phase of flying is the angle between the leg and ski. A low value of this angle allows the jumper a quick transition to a favourable position of the body and ski position. Correlation coefficients with the multi-item variable length of the jump were considerably reduced. A statistically significant value of the correlation coefficient (r=0.39; p=0.05) was found only in the sixth jump. The value of the total variance (TV=50.13%) in the first common factor was calculated and it slightly exceeded the value of 50%, thus providing the minimum criteria for a satisfactory relationship with the multi-item variable length of the jump. A significant reduction in the value of the correlation coefficients indicates a complex relationship of the performance of ski jumpers. During flight, a jumper must optimise the angle between the leg and ski, where it is important to conduct a sufficiently integrated complex system of rotation of the body and skis, which will truly take advantage of favourable aerodynamic forces during the take-off and establish the optimum position for the flight phase.

The aerodynamic aspect of take-off strongly determines the position of the skis. The research results show entirely low and statistically insignificant correlations between the multi-item variables, the angle between left and right ski, the horizontal axis, and the length of the jumps. The values of total variance in the first common factor do not reach 50%. The factor weights on the first factor are fairly homogeneous but negative. The most favourable aerodynamic position is where the skis are in a horizontal position during the early flight phase. The study of Virmavirta et al. (2005) showed that Simon Amman (Olympic champion 2002) had skis perfectly horizontally positioned during the early flight in his victories, and that this enabled him to maintain the highest possible horizontal flight speed. Displacement of the skis from that position increases the aerodynamic drag of the skis and reduces the speed of the jumper during the early flight phase. Generally, the position of the skis during the early flight phase was similar. The average value between the seven rounds of the jumps was varied by about two angular degrees. Slightly higher mean values were generally found at the position of the right ski.

No determination of significant correlation coefficients of the multi-item variable angle of hip extension and the criteria multi-item variable length of the jump was found. Based on the structure of factor weights in the first common factor, a slight positive correlation was shown. Generally, the jumpers who had longer jumps had a slightly more stretched body position at the early flight phase. A more or less stretched body position can have a negative impact on the aerodynamic aspect in the middle part of the flight. In both cases, the positive influence of aerodynamic forces and their moments can be lowered. This again underlines the aerodynamic aspect of the flight phase. For some time, the so-called flat style of flying (Flat Style) was in use. Today, this position is no longer a point of discussion, because this style is not more aerodynamically efficient and sometimes could cause problems during the technical preparation for landing. The best jumpers have average stretch angles in the hips of approximately 160 angular degrees (Schmölzer and Müller, 2005).

Correlation coefficients between the multi-item variable angle between body and left arm and the multi-item variable length of the jump were mostly not significant. Relatively homogeneous levels of factor weights on the first common factor showed a slightly negative correlation between the aforementioned multi-item variables. Slightly longer jumps were generally met with the jumpers that had smaller values of the angle between the upper body and left arm. Such integration is also understandable in terms of aerodynamics. The arms and hands must be in extended positions and close to the trunk during flight. Arm displacement away from the trunk would increase the negative aerodynamic effect of flying.

## Conclusions

On the basis of the results obtained in this research, the following conclusions can be drawn:

- The highest and most significant correlations (p<0.05) with the length of the jump were found in the multi-item variable height of flying, defined 15 m from the take-off bridge. The stability of all correlation coefficients was high and that produced the highest value of explained total variance (TV=69.13%).
- Significant (p<0.05) and relatively homogeneous correlations were found in the selected kinematic variables that indicate the aerodynamic aspect of the first phase of flying. The most important indicator of the aerodynamic aspect of flight was the angle between the body chord and the horizontal axis, which shows that body position is crucial during the flying phase. The stability of that aerodynamic factor was very high (TV=65.04%).
- The research results show low and statistically insignificant correlations between the multi-item variable angle between left and right ski and horizontal axis and the variable length of the jump. The values of the total variance on the first common factor do not reach 50%. Generally, the position of the skis during the early flight phase was relatively similar. The average value between the series of the seven jumps varied by about two angular degrees.
- No determination of significant correlation coefficients of the multi-item variable angle of hip extension and the multi-item variable length of the jump was found (p>0.05). Based on the structure of factor projections on the first common factor, a slightly positive correlation is shown. Generally, the jumpers who had longer jumps were slightly more stretched during the early flight phase.
- The successful realisation of the early flight phase was influenced by chosen kinematic multi-item variables. Each from the chosen kinematic variables has its own role in the formation of a comprehensive movement technique of the jumper. The most important for the successful realisation of the early flight phase is their optimal combination. Synergistically, they have much greater influence on the optimal composition of the ski jumping flying technique. Most kinematic factors in the early flight phase with the highest correlation validity formed a high stability structure of a ski jumping technique.

There were several limitations of this study, especially those connected to the precision of the data. A relatively large potential error in determining the chosen kinematic angle parameters could cause imprecise calculations of correlation coefficients. For this reason, the establishment of correlation validity will never be independent from the potential errors produced by the measurement procedure. The correlation validity of biomechanical early flight data could be influenced by other factors, such as the number of analysed athletes and their skill levels. With the increase of quality and homogeneity, the correlation coefficients between the mechanical parameters and the length of the jump decrease.

## Figures and Tables

**Picture 1:**
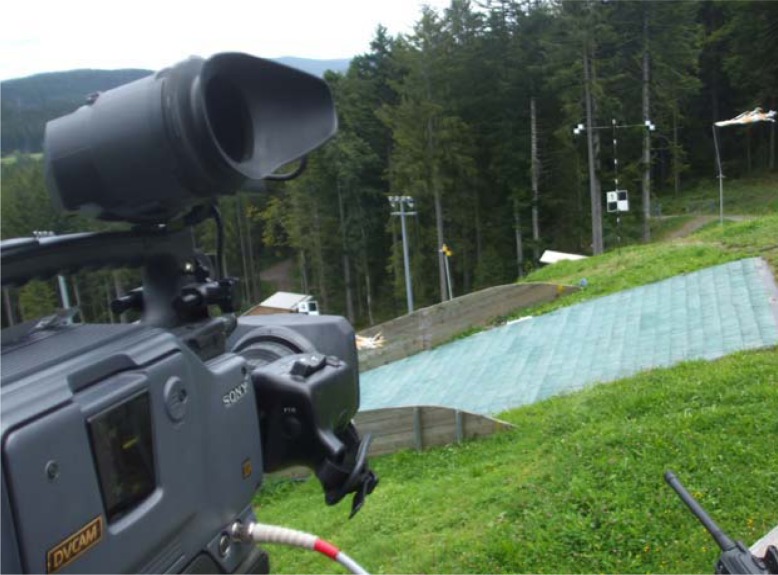
Position of camera at 15 m behind the jumping hill edge

**Picture 2:**
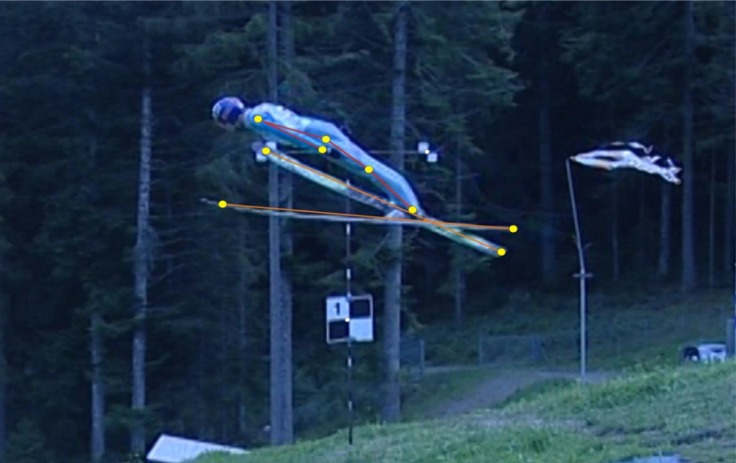
The 2-D model of nine jumper’s body and skis points used in digitising

**Table 1:** Observed variables of the ski jumpers 15m behind the jumping hill edge

**Variable**	**Mean**	**SD**	**r**	**F_1_p**	**F_2_p**	**F_CUM_**
Vertical height of flying 1 (m)	3.65	0.15	.64**	.90	−.18	.83
Vertical height of flying 2 (m)	3.66	0.18	.57**	.87	−.36	.88
Vertical height of flying 3 (m)	3.71	0.18	.72**	.87	−.37	.89
Vertical height of flying 4 (m)	3.67	0.17	.62**	.86	−.29	.82
Vertical height of flying 5 (m)	3.71	0.19	.43**	.75	−.51	.81
Vertical height of flying 6 (m)	3.67	0.16	.47**	.85	−.36	.86
Vertical height of flying 7 (m)	3.71	0.17	.65**	.92	−.22	.89
Length of jump 1 (m)	89.5	8.7		.78	.39	.76
Length of jump 2 (m)	91.7	9.2		.79	.28	.70
Length of jump 3 (m)	92.0	8.7		.88	.24	.83
Length of jump 4 (m)	91.3	7.5		.83	.44	.88
Length of jump 5 (m)	91.8	8.1		.81	.31	.75
Length of jump 6 (m)	93.7	6.9		.74	.48	.78
Length of jump 7 (m)	89.2	8.5		.78	.29	.69
% of TV				69.13	12.17	81.30
Angle between body chord and horizontal axis 1 (°)	40.1	5.7	−.47**	.85	.42	.89
Angle between body chord and horizontal axis 2 (°)	39.7	4.5	−.50**	.93	.26	.93
Angle between body chord and horizontal axis 3 (°)	39.0	4.7	−.50**	.83	.42	.87
Angle between body chord and horizontal axis 4 (°)	39.7	4.6	−.43*	.84	.47	.93
Angle between body chord and horizontal axis 5 (°)	39.8	4.4	−.40*	.84	.47	.93
Angle between body chord and horizontal axis 6 (°)	38.9	4.5	−.46**	.88	.42	.95
Angle between body chord and horizontal axis 7 (°)	39.7	5.0	−.48**	.90	.33	.92
Length of jump 1 (m)	89.5	8.7		−.74	.44	.74
Length of jump 2 (m)	91.7	9.2		−.66	.56	.74
Length of jump 3 (m)	92.0	8.7		−.80	.43	.83
Length of jump 4 (m)	91.3	7.5		−.79	.50	.87
Length of jump 5 (m)	91.8	8.1		−.71	.50	.75
Length of jump 6 (m)	93.7	6.9		−.75	.41	.73
Length of jump 7 (m)	89.2	8.5		−.71	.45	.70
% of TV				65.04	19.26	84.30
Angle between left leg and horizontal axis 1 (°)	52.1	6.5	−.46**	.82	.47	.90
Angle between left leg and horizontal axis 2 (°)	50.9	5.3	−.42*	.82	.31	.78
Angle between left leg and horizontal axis 3 (°)	50.5	5.9	−.47**	.85	.42	.89
Angle between left leg and horizontal axis 4 (°)	51.0	5.3	−.34	.81	.51	.92
Angle between left leg and horizontal axis 5 (°)	51.1	5.3	−.39*	.88	.40	.94
Angle between left leg and horizontal axis 6 (°)	50.1	5.2	−.44*	.87	.44	.95
Angle between left leg and horizontal axis 7 (°)	50.9	5.9	−.36*	.88	.38	.92
Length of jump 1 (m)	89.5	8.7		−.76	.41	.75
Length of jump 2 (m)	91.7	9.2		−.63	.59	.75
Length of jump 3 (m)	92.0	8.7		−.78	.47	.83
Length of jump 4 (m)	91.3	7.5		−.76	.54	.87
Length of jump 5 (m)	91.8	8.1		−.69	.53	.75
Length of jump 6 (m)	93.7	6.9		−.75	.43	.74
Length of jump 7 (m)	89.2	8.5		−.66	.52	.71
% of TV				61.88	21.59	83.47

Mean - the average values, SD - standard deviations, r – linear Pearson coefficient of correlations between more items variables and length of the jumps (r), F_1_p - first factor projections of manifest variables, F_2_p - second factor projections of manifest variables, **F**_CUM_ - cumulative values of factor loadings, % of TV – percent of total explained variance,

*. Correlation is significant at the 0.05 level (2-tailed),

**. Correlation is significant at the 0.01 level (2-tailed)

**Table 2:** Observed variables of the ski jumpers 15m behind the jumping hill edge

**Variable**	**Mean**	**SD**	**r**	**F_1_p**	**F_2_p**	**F _CUM_**
Angle of hip extension 1 (°)	150.6	7.0	.21	.42	.70	.67
Angle of hip extension 2 (°)	153.1	6.7	.03	.45	.30	.29
Angle of hip extension 3 (°)	150.7	6.6	.25	.67	.59	.80
Angle of hip extension 4 (°)	152.9	6.6	−.05	.36	.86	.86
Angle of hip extension 5 (°)	152.0	6.9	.33	.72	.55	.82
Angle of hip extension 6 (°)	152.2	5.2	.12	.48	.79	.85
Angle of hip extension 7 (°)	152.0	6.1	.21	.42	.77	.77
Length of jump 1 (m)	89.5	8.7		.85	−.23	.78
Length of jump 2 (m)	91.7	9.2		.68	−.52	.73
Length of jump 3 (m)	92.0	8.7		.78	−.46	.83
Length of jump 4 (m)	91.3	7.5		.82	−.43	.86
Length of jump 5 (m)	91.8	8.1		.74	−.46	.76
Length of jump 6 (m)	93.7	6.9		.80	−.32	.75
Length of jump 7 (m)	89.2	8.5		.65	−.56	.74
% of TV				42.71	32.35	75.06
Angle between upper body and horizontal axis 1 (°)	21.0	5.5	−.22	**.72**	.38	.66
Angle between upper body and horizontal axis 2 (°)	21.7	4.9	−.43*	**.72**	.33	.62
Angle between upper body and horizontal axis 3 (°)	19.5	4.5	−.26	**.67**	.63	.86
Angle between upper body and horizontal axis 4 (°)	21.5	5.2	−.36*	**.82**	.46	.88
Angle between upper body and horizontal axis 5 (°)	21.2	4.5	−.12	**.61**	.69	.85
Angle between upper body and horizontal axis 6 (°)	20.4	4.4	−.27	**.80**	.51	.89
Angle between upper body and horizontal axis 7 (°)	21.2	3.9	−.54**	**.82**	.39	.83
Length of jump 1 (m)	89.5	8.7		−**.62**	.62	.77
Length of jump 2 (m)	91.7	9.2		−**.70**	.48	.72
Length of jump 3 (m)	92.0	8.7		−**.79**	.45	.83
Length of jump 4 (m)	91.3	7.5		−**.78**	.51	.87
Length of jump 5 (m)	91.8	8.1		−**.72**	.49	.75
Length of jump 6 (m)	93.7	6.9		−**.70**	.50	.74
Length of jump 7 (m)	89.2	8.5		−**.75**	.39	.71
% of TV				**53.69**	24.79	78.48
Angle between left skis and left legs (°)	38.8	7.4	−.33	**.74**	.45	.75
Angle between left skis and left legs (°)	38.7	8.1	−.30	**.78**	.47	.83
Angle between left skis and left legs (°)	38.8	7.3	−.13	**.72**	.54	.82
Angle between left skis and left legs (°)	37.5	7.8	−.10	**.65**	.56	.74
Angle between left skis and left legs (°)	36.7	6.7	−.06	**.66**	.60	.79
Angle between left skis and left legs (°)	37.4	8.3	−.39*	**.78**	.50	.86
Angle between left skis and left legs (°)	36.8	7.2	−.26	**.76**	.46	.79
Length of jump 1 (m)	89.5	8.7		−**.69**	.53	.75
Length of jump 2 (m)	91.7	9.2		−**.66**	.55	.73
Length of jump 3 (m)	92.0	8.7		−**.69**	.59	.83
Length of jump 4 (m)	91.3	7.5		−**.67**	.65	.88
Length of jump 5 (m)	91.8	8.1		−**.70**	.51	.75
Length of jump 6 (m)	93.7	6.9		−**.71**	.49	.74
Length of jump 7 (m)	89.2	8.5		−**.68**	.48	.69
% of TV				**50.13**	28.06	78.19

*Mean - the average values, SD - standard deviations, r – linear Pearson coefficient of correlations between more items variables and length of the jumps (r), F_1_p - first factor projections of manifest variables, F_2_p - second factor projections of manifest variables, **F**_CUM_ - cumulative values of factor loadings,* % *of TV – percent of total explained variance,*

*. Correlation is significant at the 0.05 level (2-tailed),

**. Correlation is significant at the 0.01 level (2-tailed)

**Table 3:** Observed variables of the ski jumpers 15m behind the jumping hill edge

**Variable**	**Mean**	**SD**	**r**	**F_1_p**	**F_2_p**	**F _CUM_**
Angle between left skis and horizontal axis (°)	14.8	7.3	−.15	−.53	.59	.62
Angle between left skis and horizontal axis (°)	14.1	6.0	.01	−.52	.71	.78
Angle between left skis and horizontal axis (°)	13.2	6.3	−.28	−.61	.63	.77
Angle between left skis and horizontal axis (°)	15.5	6.5	−.19	−.58	.56	.66
Angle between left skis and horizontal axis (°)	15.9	5.2	−.22	−.63	.61	.77
Angle between left skis and horizontal axis (°)	14.3	6.5	.34	−.54	.69	.76
Angle between left skis and horizontal axis (°)	15.5	7.2	.16	−.64	.57	.73
Length of jump 1 (m)	89.5	8.7		.78	.40	.76
Length of jump 2 (m)	91.7	9.2		.62	.59	.74
Length of jump 3 (m)	92.0	8.7		.78	.47	.83
Length of jump 4 (m)	91.3	7.5		.81	.48	.88
Length of jump 5 (m)	91.8	8.1		.62	.62	.77
Length of jump 6 (m)	93.7	6.9		.67	.54	.74
Length of jump 7 (m)	89.2	8.5		.66	.51	.69
% of TV				42.00	33.05	75.05
Angle between right skis and horizontal axis 1 (°)	20.3	6.6	−.36*	−.76	.37	.71
Angle between right skis and horizontal axis 2 (°)	20.0	4.9	.02	−.51	.64	.67
Angle between right skis and horizontal axis 3 (°)	20.3	6.7	−.15	−.52	.64	.68
Angle between right skis and horizontal axis 4 (°)	21.0	6.3	−.11	−.55	.62	.69
Angle between right skis and horizontal axis 5 (°)	21.3	6.1	−.30	−.61	.58	.72
Angle between right skis and horizontal axis 6 (°)	20.8	4.4	.08	−.57	.63	.73
Angle between right skis and horizontal axis 7 (°)	20.7	6.9	.08	−.74	.41	.71
Length of jump 1 (m)	89.5	8.7		.71	.49	.75
Length of jump 2 (m)	91.7	9.2		.69	.51	.73
Length of jump 3 (m)	92.0	8.7		.80	.44	.83
Length of jump 4 (m)	91.3	7.5		.81	.47	.87
Length of jump 5 (m)	91.8	8.1		.75	.42	.74
Length of jump 6 (m)	93.7	6.9		.85	.23	.79
Length of jump 7 (m)	89.2	8.5		.65	.55	.72
% of TV				47.34	26.51	73.85
Angle between upper body and left arm 1 (°)	5.8	9.7	.18	**.53**	.62	.67
Angle between upper body and left arm 2 (°)	7.6	9.0	−.08	**.70**	.56	.80
Angle between upper body and left arm 3 (°)	5.2	7.1	−.20	**.70**	.63	.88
Angle between upper body and left arm 4 (°)	8.8	8.2	−.25	**.79**	.53	.90
Angle between upper body and left arm 5 (°)	6.9	8.2	−.38*	**.74**	.48	.78
Angle between upper body and left arm 6 (°)	8.2	8.4	−.14	**.71**	.50	.76
Angle between upper body and left arm 7 (°)	8.4	7.7	−.41*	**.81**	.40	.81
Length of jump 1 (m)	89.5	8.7		**−.54**	.70	.78
Length of jump 2 (m)	91.7	9.2		**−.58**	.63	.73
Length of jump 3 (m)	92.0	8.7		**−.73**	.54	.83
Length of jump 4 (m)	91.3	7.5		**−.71**	.61	.87
Length of jump 5 (m)	91.8	8.1		**−.73**	.48	.76
Length of jump 6 (m)	93.7	6.9		**−.59**	.63	.74
Length of jump 7 (m)	89.2	8.5		**−.71**	.45	.71
% of TV				**47.47**	31.31	78.78

Mean - the average values, SD - standard deviations, r – linear Pearson coefficient of correlations between more items variables and length of the jumps (r), F_1_p - first factor projections of manifest variables, F_2_p - second factor projections of manifest variables, **F**_CUM_ - cumulative values of factor loadings, % of TV – percent of total explained variance,

*. Correlation is significant at the 0.05 level (2-tailed)
